# An observational pilot study: Prevalence and cost of high frequency emergency department users at Örebro University Hospital, Sweden

**DOI:** 10.1371/journal.pone.0274622

**Published:** 2022-09-15

**Authors:** Ivy Cheng, Jonas Andersson, Christer Lundqvist, Lisa Kurland

**Affiliations:** 1 Division of Emergency Medicine, University of Toronto, Toronto, Ontario, Canada; 2 Sunnybrook Health Sciences Center, Toronto, Ontario, Canada; 3 School of Medical Sciences, Örebro University, Örebro, Sweden; 4 Centre for Clinical Research Sörmland/Uppsala University, Mälarsjukhuset, Eskilstuna, Sweden; 5 Health and Medical Care Administration, Örebro County Council, Örebro, Sweden; Northumbria University, UNITED KINGDOM

## Abstract

**Background:**

There is little research on high frequency emergency department users (HEDU) in Sweden. We aim to determine the prevalence and costs of HEDU compared to non-HEDU at Örebro University Hospital (ÖUH). Additionally, we will determine the factors and outcomes associated with being a HEDU.

**Methods:**

This was a retrospective, observational cohort study of ED patients presenting to ÖUH, Sweden between 2018–19. Analyses used electronic registry, ambulance, and cost data. The definition for HEDU was ≥4 visits/year. HEDUs were categorized further into Repeat, High and Super HEDU with 4–7, 8–18 and ≥19 visits/year, respectively. We used multivariable logistic regression to determine the adjusted odds ratios for factors and outcomes between HEDU and non-HEDU.

**Findings:**

Of all ÖUH ED patients, 6.1% were HEDU and accounted for 22.4% of ED visits and associated costs. Compared to the mean cost of non-HEDU, the Repeat, High and Super HEDU were more costly by factors of 4, 8 and 27, respectively. The HEDUs were more likely to be male, self-referred, present with abdominal pain, arrive by ambulance, at night and from the Örebro municipal region. Super HEDU were more likely to be of adult age and assigned lower acuity scores. HEDU were more likely to be directed to the surgical zone, less likely to receive radiologic imaging or achieve a 4-hr time target. In contrast to the Repeat and High HEDU, Super HEDU were less likely to be admitted, but more likely to leave without being seen.

**Conclusion:**

ÖUH has a HEDU population with associated factors and outcomes. They account for a substantial proportion of ED costs compared to non-HEDU.

## Introduction

According to the Organization for Economic Co-operation and Development (OECD), healthcare spending will increase by an average of 2.7% above inflation per year by 2030 for OECD countries [1]. Consequently, there is increasing focus on providing high-quality and cost-effective healthcare. The OECD has recommended more effective healthcare spending by using cheaper generic medications, shifting work from physicians to lower-paying mid-level providers, and decreasing nosocomial infections [1]; however, there has been little mention of the high-cost users of health care. Although high-cost users are a small portion of the population, they account for a disproportionate amount of health care costs [2] with continuing and unmet needs [3]. A 2018 systematic review found that the top 10%, 5% and 1% high-cost users accounted for 68%, 55% and 24% of annual costs, respectively [3]. They were more likely to be older, complex, at end of life, with high levels of chronic and mental illness; however, other characteristics, such as socioeconomic deprivation, ethnicity, physician trust and health behaviors were not universally shared [3]. High-cost users include the high emergency department user (HEDU) [3,4] with frequent, costly [5] and likely avoidable emergency visits [3]. The definition of the HEDU is not universally agreed upon [6], but many international studies use ≥4 ED visits/year [5]. HEDUs share some high-cost user characteristics, such as mental or chronic illness; but did not share others, such as ethnicity or insurance status, which seemed dependent on jurisdiction [5]. So, to achieve high-quality, cost-effective care, healthcare systems need to focus and improve care for these vulnerable populations with a tailored approach to their jurisdiction [3].

According to a recent systematic review, most HEDU research was American with only a single Swedish study [5]. Between 1990 and 2014, there were a handful of Swedish studies in Stockholm City or Huddinge which examined HEDU risk factors, resource consumption, outcomes and interventions [7–11]. Additionally, there are no published studies on the costs of Swedish HEDUs. Consequently, there is a knowledge gap of Swedish HEDUs including associated costs. We would like to determine if it is feasible to capture HEDU data at a local Swedish hospital, Örebro University Hospital (ÖUH). If achieved, HEDU data collection could be expanded regionally and nationally. Since this is the first Swedish study of its kind, it is a pilot study.

Our primary aim is to determine the prevalence and costs of HEDU (≥4 visits/year) compared to non-HEDU at ÖUH. Our secondary aim is to determine the factors and outcomes associated with being a HEDU using the electronic health record.

## Methods

### Study design and setting

This was a retrospective, observational cohort study of ED patients presenting to ÖUH.

Sweden has a universal health care system. Hospital and physician payments are subsidized, with only a small part of the funding coming from patient fees (100–400 SEK (12.18–48.73 USD) [12] January 1, 2018) depending on type of visit and the rest funded by taxes. There is a ceiling to the annual cost of health care for each citizen equivalent to 1150 SEK (140.11 USD) [13]. Örebro is the sixth largest city in Sweden with a population of 124,027 [14], catchment area of 302 252 (2019) [15] and 60 general practitioners per 100 000 population. ÖUH is an academic hospital. Its adult-pediatric ED receives >60,000 visits per year and is a trauma, interventional cardiology, stroke, thoracic and neurosurgical center. Upon arrival, a pre-triage nurse records the patient’s chief complaint and performs a quick assessment. If the chief complaint is a psychiatric, gynecological, eye or ear-nose-and-throat, the patient is re-directed to a separate specialty-specific department outside the ED. Minor orthopedic injuries are also re-directed to a separate department during office hours. The main triage assessment is conducted by a registered nurse using the Swedish acuity score: Rapid Emergency Triage and Treatment System (RETTS). Once registered in the ED, patients are triaged into different ED zones, such as surgical, medical, or pediatric (<18 years old) and can, if needed, be transferred from one zone to another. Each zone transfer is considered a separate ED registration. ED staffing of doctors is predominantly rotating junior physicians representing the different specialties, as well as a smaller number of emergency consultants. Nurses have the capacity to independently assess and disposition patients. The ED has no short-stay unit. The hospital had on average 402 beds during the study period.

### Patient population, data sources and objectives

The patient population was ED patients visiting ÖUH between January 1, 2018, to December 31, 2019. We used three electronic data sources: the Örebro ED registry, ambulance data and regional costing ledger. The Örebro ED Registry collected patient demographics, presenting complaint, acuity score, allocated ED zone (eg. medical, pediatrics, surgical), number of transfers between zones, provider type, disposition, and time markers (eg. discharge time). Ambulance data recorded patient information, including time of ambulance arrival. We used patient identifiers and ED encounter numbers to combine the Örebro ED Registry, costing ledger and ambulance data into a single dataset. The costing ledger included costs associated with the ED visit (eg. provider, radiology, or drug costs). In Sweden, there is a national standard for calculating the cost per patient. The base cost is determined by the type of personnel (doctor or nurse) caring for the patient. If the patient receives further resources, such as radiology, pathology, or laboratory, they are added to the base cost. Ambulance costs are not included. All assigned costs are validated by the Örebro economic department before they become official nationally. Research ethics was approved by the Swedish Ethical Review Authority (Registration number: 2020–01614).

The primary objective was the prevalence of ÖUH HEDU and associated total and mean costs per visit and patient between January 1, 2018, and December 31, 2019. Secondary objectives were factors and outcomes associated with HEDU visits from the electronic health record.

### Data analysis

**Fig 1:**
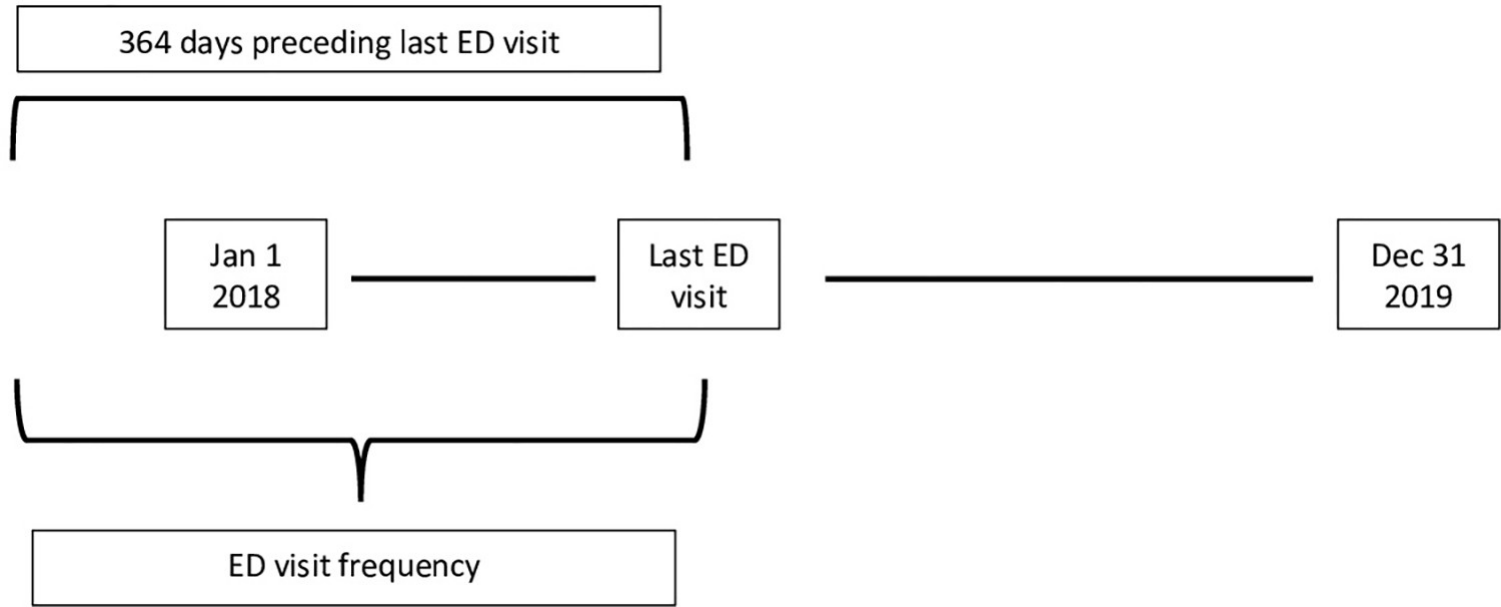
Method to determine emergency department (ED) visit frequency for HEDUs.

Because zone transfers were registered separately, only index ED registrations were counted. Duplications and registrations with missing cost data were excluded. Following international literature [5], we defined HEDU as ≥4 ED visits per year. Using methods by Doupe et al. [16], we calculated visit frequency by counting backwards from the patient’s last ED visit within the preceding year (Fig 1). Referencing Liu et al. [17], we defined Repeat, High and Super HEDU as 4–7, 8–18 and ≥19 visits over one year. We collected demographics, acuity score, municipality code, referral source, arrival mode, arrival time, presenting complaint, assigned zone, number of zone transfers per visit, radiology consumption, disposition, and ED length-of-stay (LOS) by HEDU type. Using the standard age structure [18], we organized age into three groups: children (0-14yo), adults (15-64yo) and elderly (≥65 yo). RETTS is a 5-tiered triage system, where the highest acuity level is red, which we have depicted as 1 [19]. For simplicity and consistency with emergency medicine literature, we combined RETTS into two categories: High (1–2) and Low (3–5) acuity. We categorized patients by disposition: admission, discharge, death or left-without-being-seen. Discharged patients were grouped into low and high acuity. EDLOS was the duration between ED arrival and discharge (i.e. door to door time). Next, organized by HEDU type and from a public healthcare payer perspective, we determined the total and mean cost by visit and patient. Then, we determined patient factors associated with HEDU type. Finally, we determined the relationship between HEDU type and outcomes of assigned zone, radiology consumption, admission and EDLOS time targets. Although the 4-hr rule has become obsolete in Sweden, other jurisdictions, such as the United Kingdom, continue to use the rule. Consequently, we applied the United Kingdom EDLOS 4-hr target [20].

We presented descriptive metrics by percentages and means with 95% percent confidence intervals. For costs, we converted Swedish krona to USD based on the January 1, 2018, conversion rate. At that time, 1 SEK was 0.121833 USD (1 USD = 8.207955 SEK) [12]. Radiology and provider costs were main contributors to total costs, so all three costs were reported. For the binary outcome of HEDU type vs. non-HEDU, we used multivariable logistic regression (alpha level of 0.05) to determine the adjusted odds ratios and 95% confidence intervals for predictor factors. Because there were three types of HEDU (Repeat, High, Super), we performed three separate regressions. For selected outcomes, we also used multivariable logistic regression (alpha level of 0.05) to determine odds ratios and 95% confidence intervals for the covariate of HEDU type (Repeat, High, Super). We used Matlab [21] to combine the Örebro ED Registry and ambulance data. Analyses were performed using Stata 15 [22] and Excel [23].

## Results

**Fig 2:**
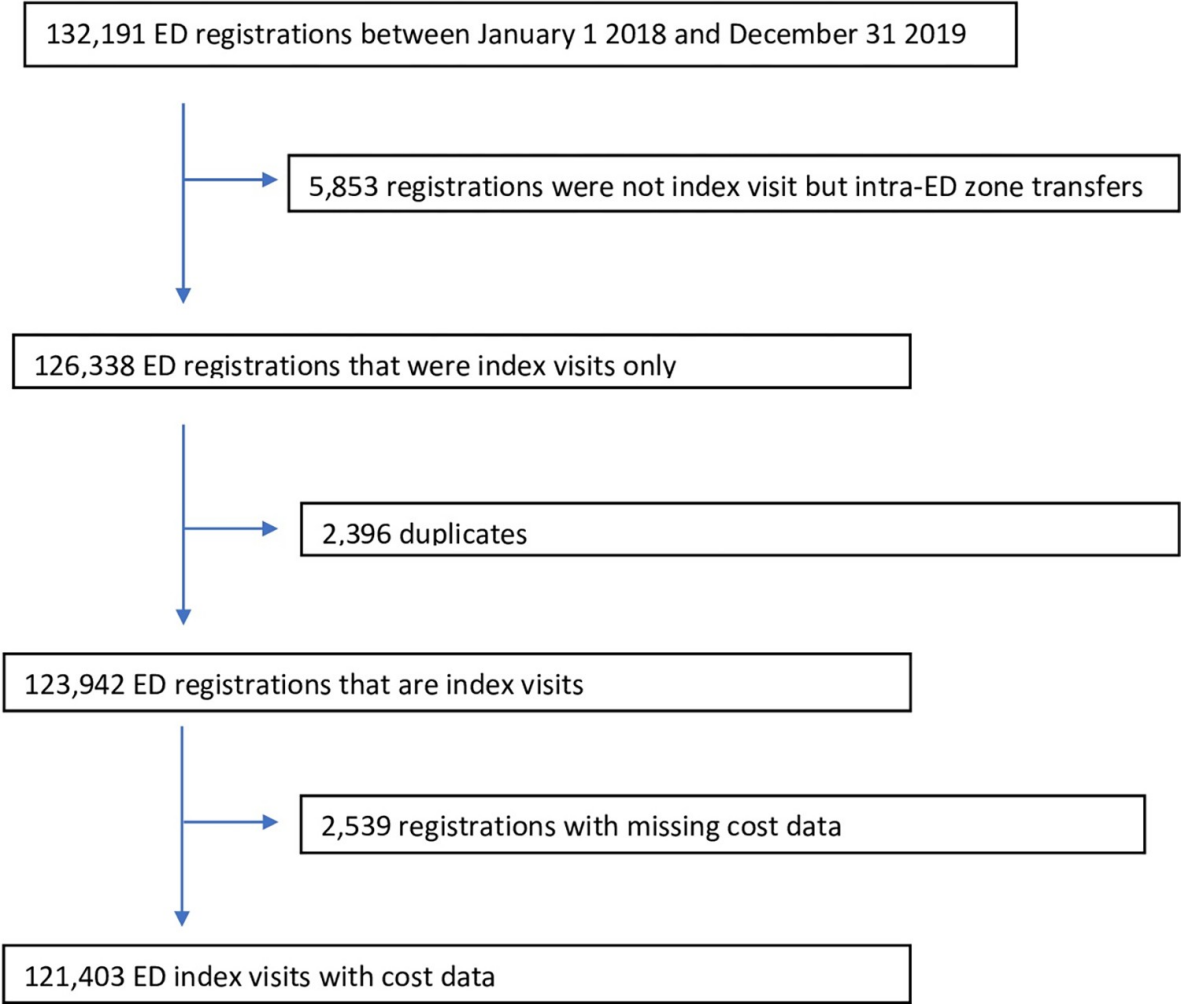
Flow diagram of Örebro ED registration data.

Between January 1, 2018 to December 31, 2019, there were 132,191 ED registrations. We excluded non-index registrations by the same patient (4.4%), duplications (1.9%) or missing cost data (2.0%). Consequently, 121,403 index ED visits remained attended by 69,984 patients (Fig 2).

Mean age was 41.4 with 23.9% and 27.9% of 121,403 visits in the children and elderly group, respectively. We found that 50.9% of patients were male, and 21.5% presented with an acuity score of RETTS1-2. Of all the visits, 91.8% were self-referred, 19.0% arrived by ambulance and 65.2% from Örebro municipal region. The percentage of visits during day, evening and night hours were 45.3%, 42.3% and 12.5%, respectively, with the most common presenting complaint of abdominal pain, followed by chest pain and fever. Once in the ED, 28.6%, 28.1%, 15.9%, 11.2% and 5.1% were assigned to the surgical, medical, pediatrics, orthopedics, and neurological zones, respectively. Less than 4% of patients were transferred internally, with little difference (0.3%) between HEDU and non-HEDU. About 21.7% received radiological testing. For disposition, 26.5% were admitted, 70.8% discharged, 2.7% left without being seen and 0.1% died. Median EDLOS was 3.1 hrs with the shortest being 0.4hrs (death) and longest at 3.4hrs (admissions and discharged high acuity). Although 6.1% of ED patients were HEDU, they accounted for 22.4% of ED visits. Of all the ED patients, 5.3%, 0.7% and 0.1% were Repeat (4–7 visits), High (8–18 visits) and Super (> = 19 visits) HEDU, respectively. The Super HEDU group did not have any children but did have the greatest proportion of adults (79.0%). In contrast, the Repeat HEDU group had the greatest proportion of children (24.4%) and elderly (38.0%) (Table 1).

**Table 1:** Characteristics of January 1, 2018 –December 31, 2019 Örebro ED visits.

Demographics	All-comers	Non-HEDU 1–3 visits	HEDU > = 4 visits			
				Repeat HEDU 4–7 visits	High HEDU 8–18 visits	Super HEDU > = 19 visits
N visits (%)	121,403 (100.0%)	94,185 (77.5%)	27,218 (22.4%)	19,551 (16.1%)	5,878 (4.8%)	1,789 (1.5%)
N patients (%)	69,984 (100.0%)	65,742 (93.9%)	4,242 (6.1%)	3.715 (5.3%)	485 (0.7%)	42 (0.1%)
Age: Mean (95% CI)	41.4 [1.0, 86.0]	40.0 [1.0, 85.0]	46.2 [1.0, 88.0]	45.4 [1.0, 88.0]	47.1 [1.0, 86.0]	51.6 (24.0, 82.0]
Age Group Children: 0-14yo Adult: 15–64 yo Elderly: > = 65 yo	29,049 (23.9%) 58,454 (48.2%) 33,870 (27.9%)	23,190 (24.6%) 46,951 (49.9%) 24,014 (35.5%)	5,859 (21.5%) 11,503 (42.3%) 9,856 (36.2%)	4,775 (24.4%) 7,347 (37.6%) 7,429 (38.0%)	1,084 (18.4%) 2,742 (46.6%) 2,052 (34.9%)	0 (0.0%) 1,414 (79.0%) 375 (21.0%)
Sex Male Female Other	59,608 (50.9%) 61,782 (49.1%) 13 (0.0%)	47,512 (50.4%) 46,660 (49.5%) 13 (0.0%)	14,270 (52.4%) 12,948 (47.6%) 0 (0.0%)	10,176 (52.0%) 9,375 (48.0%) 0 (0.0%)	3,118 (53.0%) 2,760 (47.0%) 0 (0.0%)	976 (54.6%) 813 (45.4%) 0 (0.0%)
RETTS Acuity Score 1 2 3 4 5 Missing	5,019 (4.1%) 21,108 (17.4%) 55,694 (45.9%) 22,272 (18.3%) 11,203 (9.2%) 6,107 (5.0%)	3,681 (3.9%) 15,363 (16.3%) 42,878 (45.5%) 17,441 (18.5%) 9,922 (10.5%) 4,870 (5.2%)	1,338 (4.9%) 5,745 (21.1%) 12,816 (47.1%) 4,831 (17.7%) 1,281 (4.7%) 1,207 (4.4%)	970 (5.0%) 4,053 (20.7%) 9,229 (47.2%) 3,522 (18.0%) 983 (5.0%) 794 (4.1%)	316 (5.4%) 1,339 (22.8%) 2,705 (46.0%) 991 (16.9%) 222 (3.8%) 305 (5.2%)	52 (2.9%) 353 (19.7%) 882 (49.3%) 318 (17.8%) 76 (4.2%) 108 (6.0%)
Acuity High: RETTS 1–2 Low: RETTS 3–5 Missing	26,127 (21.5%) 86,169 (73.4%) 6,107 (5.0%)	19.044 (20.2%) 70,241 (74.6%) 4,900 (5.2%)	7.083 (26.0%) 18.928 (69.5%) 1,207 (4.4%)	5.023 (25.7%) 13.734 (70.2%) 794 (4.1%)	1,655 (28.2%) 3.918 (66.7%) 305 (5.2%)	405 (22.6%) 1,276 (71.3%) 108 (6.0%)
Municipal Region 1880 (Örebro) 1881 (Kumla) 1861 (Hallsberg) 1882 (Askersund) 1814 (Lekeberg) 1885 (Lindesberg) 1884 (Nora) 1883 (Karlskoga)	79,088 (65.2%) 10,147 (8.4%) 7,966 (6.6%) 4,900 (4.0%) 3,352 (2.8%) 2,803 (2.3%) 1,673 (1.4%) 1,504 (1.2%)	59,776 (63.5%) 7,876 (8.4%) 6,003 (6.4%) 3,951 (4.2%) 2,609 (2.8%) 2,348 (2.5%) 1318 (1.4%) 1379 (1.5%)	19,312 (71.0%) 2271 (8.3%) 1963 (7.2%) 949 (3.5%) 743 (2.7%) 455 (1.7%) 355 (1.3%) 125 (0.5%)	13,629 (69.7%) 1,748 (8.9%) 1,461 (7.5%) 740 (3.8%) 556 (2.8%) 363 (1.9%) 185 (1.0%) 124 (0.6%)	4,246 (72.2%) 435 (7.4%) 402 (6.8%) 201 (3.4%) 142 (2.4%) 92 (1.6%) 119 (2.0%) 0 (0.0%)	1,437 (80.3%) 88 (4.9%) 100 (5.6%) 8 (0.5%) 45 (2.5%) 0 (0.0%) 51 (2.9%) 1 (0.1%)
Municipal Region 1880 (Örebro) Not 1880 (Outside Örebro)	79,088 (65.2%) 42,315 (34.9%)	59,776 (63.5%) 34,409 (36.5%)	19,312 (71.0%) 7.906 (29.0%)	13,629 (69.7%) 5,922 (30.3%)	4,246 (72.2%) 1,632 (27.8%)	1,437 (80.3%) 352 (19.7%)
Referred From: Self Outside Clinic	111,411 (91.8%) 9,992 (8.2%)	85,682 (91.0%) 8,503 (9.0%)	25,729 (94.5%) 1,489 (5.5%)	18,295 (93.6%) 1,256 (6.4%)	5,675 (96.6%) 203 (3.5%)	1,759 (98.3%) 30 (1.7%)
Arrival Mode Walk-In Ambulance	98,291 (81.0%) 23,112 (19.0%)	77,455 (82.2%) 16,730 (17.8%)	20,836 (76.6%) 6,382 (23.4%)	15,124 (77.4%) 4,427 (22.6%)	4,448 (75.5%) 1,430 (24.3%)	1,246 (70.7%) 525 (29.3%)
Arrival Time: Day (8:00–15:59) Evening (16:00–23:59) Night (00:00–07:59)	54,985 (45.3%) 51,306 (42.3%) 15,112 (12.5%)	42,572 (45.2%) 40,435 (42.9%) 11,178 (11.9%)	12,413 (45.6%) 10,871 (39.9%) 3,934 (14.5%)	9,186 (47.0%) 7,714 (39.5%) 2,651 (13.6%)	2,583 (43.9%) 2,342 (39.8%) 953 (16.2%)	644 (36.0%) 815 (45.6%) 220 (18.4%)
Presenting Complaint (Top 4): Abdominal Pain Chest Pain Fever Difficulty Breathing	19,022 (16.0%) 7,786 (6.6%) 7,106 (6.0%) 5,947 (5.0%)	13,750 (14.9%) 6,234 (6.8%) 5,289 (5.7%) 4,043 (4.4%)	5,272 (19.8%) 1,552 (5.8%) 1,817 (6.8%) 1,904 (7.1%)	3,425 (17.9%) 1,079 (5.6%) 1,440 (7.5%) 1,410 (7.4%)	1,103 (19.2%) 398 (6.9%) 371 (6.5%) 431 (7.5%)	744 (42.8%) 75 (4.3%) 6 (0.3%) 63 (3.6%)
Emergency Department Zone: Surgical Medical Pediatrics Orthopedics Neurology Ear, Nose and Throat Infectious Disease Urology Hand Surgery Emergency Maxillofacial	34,745 (28.6%) 34,064 (28.1%) 19,275 (15.9%) 13,636 (11.2%) 6,186 (5.1%) 4,919 (4.1%) 3,550 (2.9%) 2,525 (2.1%) 1,692 (1.4%) 616 (0.5%) 179 (0.2%)	26,076 (27.7%) 25,802 (27.4%) 14,517 (15.4%) 12,103 (12.9%) 4,976 (5.3%) 4,198 (4.5%) 2,776 (3.0%) 1,572 (1.7%) 1,518 (1.6%) 479 (0.5%) 155 (0.2%)	8,669 (31.9%) 8,262 (30.4%) 4,758 (17.5%) 1,533 (5.6%) 1,210 (4.5%) 721 (2.7%) 774 (2.6%) 953 (3.5%) 174 (0.6%) 137 (0.5%) 24 (0.1%)	5.783 (29.6%) 5,840 (29.9%) 3,815 (19.5%) 1,198 (6.1%) 879 (4.5%) 548 (2.8%) 595 (3.0%) 641 (3.3%) 130 (0.7%) 100 (0.5%) 19 (0.1%)	1,895 (32.3%) 1,909 (32.5%) 943 (16.0%) 236 (4.0%) 235 (4.0%) 141 (2.4%) 163 (2.8%) 284 (4.8%) 43 (0.7%) 25 (0.4%) 4 (0.1%)	991 (55.4%) 513 (28.7%) 0 (0.0%) 99 (5.5%) 96 (5.4%) 32 (1.8%) 16 (0.9%) 28 (1.6%) 1 (0.1%) 12 (0.7%) 1 (0.1%)
Zone Transfers 0 1 2 3 4	116,925 (96.3%) 4,121 (3.4%) 330 (0.3%) 23 (0.0%) 4 (0.0%)	90,777 (96.4%) 3,115 (3.3%) 271 (0.3%) 19 (0.0%) 3 (0.0%)	26,148 (96.1%) 1,006 (3.7%) 59 (0.2%) 4 (0.0%) 1 (0.0%)	18,767 (96.0%) 742 (3.8%) 38 (0.2%) 4 (0.0%) 0 (0.0%)	5,640 (96.0%) 220 (3.7%) 17 (0.3%) 0 (0.0%) 1 (0.0%)	1,741 (97.3%) 44 (2.5%) 4 (0.2%) 0 (0.0%) 0 (0.0%)
Radiology Yes No	26,378 (21.7%) 95.025 (78.3%)	21,751 (23.1%) 72,434 (76.9%)	4,627 (17.0%) 22,591 (83.0%)	3,561 (18.2%) 15,990 (81.8%)	881 (15.0%) 4,997 (85.0%)	185 (10.3%) 1,604 (89.7%)
Disposition Discharged Low Acuity (RETTS 3–5) Discharged High Acuity (RETTS 1–2) Admitted LWBS Died	74,325 (61.2%) 11,584 (9.5%) 32,122 (26.5%) 3,248 (2.7%) 112 (0.1%)	59,301 (63.0%) 8,584 (9.1%) 23,788 (25.3%) 2,403 (2.6%) 100 (0.1%)	15,024 (55.2%) 3,000 (11.0%) 8,334 (30.6%) 845 (3.1%) 12 (0.0%)	10,806 (55.3%) 2,096 (10.7%) 6,110 (31.3%) 526 (2.7%) 10 (0.1%)	3,183 (54.2%) 684 (11.6%) 1,835 (31.2%) 175 (3.0%) 1 (0.0%)	1,035 (57.9%) 220 (12.3%) 389 (21.7%) 144 (8.0%) 1 (0.1%)
EDLOS: Median [IQR] Discharged Low Acuity Discharged High Acuity Admitted LWBS Died	3.0hrs [1.8, 4.5] 3.4hrs [2.3, 4.8] 3.4hrs [2.2, 5.0] 2.8hrs [1.6, 4.2] 0.4hrs [0.2, 0.8]	2.9hrs [1.7, 4.4] 3.3hrs [2.3, 4.7] 3.3hrs [2.1, 4.9] 2.8hrs [1.7, 4.3] 0.4hrs [0.2, 0.7]	3.2hrs [2.0, 4.8] 3.5hrs [2.4, 4.9] 3.5hrs [2.3, 5.1] 2.7hrs [1.6, 4.2] 0.6hrs [0.2, 1.1]	3.2hrs [1.9, 4.8] 3.5hrs [2.4, 4.9] 3.5hrs [2.3, 5.0] 2.8hrs [1.6, 4.2] 0.6hrs [0.2, 1.2]	3.2hrs [2.0, 4.9] 3.5hrs [2.4, 5.0] 3.4hrs [2.3, 5.1] 2.4hrs [1.5, 3.7] 1.0hrs [1.0, 1.0]	3.7hrs [2.2, 5.3] 3.6hrs [2.7, 5.0] 3.7hrs [2.4, 5.4] 3.2hrs [1.6, 4.4] 0.0hrs [0.0, 0.0]

IQR (Interquartile range); EDLOS (Emergency Department Length of Stay).

The 2018 and 2019 physician costs were 2726 SEK (332.12 USD) and 2872 SEK (349.90 USD), respectively, whereas 2018 and 2019 nursing (as independent provider) costs were 2261 SEK (275.46 USD) and 2346 SEK (285.82 USD), respectively. HEDU visits (97,527,226.48 SEK (11,882,034.58 USD)) accounted for 22.0% of total ED costs (442,520,842.74 SEK (53,913,641.83 USD)). Similarly, HEDU visits accounted for 22.4% and 19.8% ED provider and radiology costs, respectively. The mean cost per HEDU visit (3583.19 SEK (436.55USD)) and Super HEDU visit (3304.73 SEK (402.63 USD)) was less than the non-HEDU visit (3662.94 SEK (446.27 USD)). Radiology cost per HEDU visit was less than non-HEDU; however, provider cost was similar across all visits. The mean cost per HEDU patient (22,990.86 SEK (2801.05 USD)) was four times more costly than the non-HEDU patient (5247.69 SEK (639.34 USD)). For the Repeat, High and Super HEDU patient, the median number of visits per patient was 5, 12 and 41. Compared to the mean cost per non-HEDU patient (5247.69 SEK (639.34 USD)), the Repeat, High and Super HEDU were more costly by factors of 4 (18,981.59 SEK (2,312.58 USD)), 8 (43,502.00 SEK (5,299.98 USD)) and 27 (104,765.70 SEK (17,149.91 USD)), respectively. The provider cost per patient for Repeat, High and Super HEDU was 4, 8, and 30 times more costly than the non-HEDU patient, and the radiology cost per patient was 3, 7 and 16 times more costly than the non-HEDU patient, respectively. The maximum number of visits over one year was 190 for a single HEDU patient. Over two years, this single patient had 248 visits (58 (2018), 190 (2019)) with a total cost of 722,513.90 SEK (88,026.04 USD) (162,036.00 SEK (19,741.33 USD)(2018), 560,477.90 SEK (68,284.70 USD) (2019)). Of the total cost, 97.4% was provider costs (703,788.00 SEK (85,744.60 USD). Although the cost per Super HEDU visit was 9.8% less (-358.21 SEK (-43.64 USD)) than the non-HEDU visit, this single patient was 45 and 155 times more costly than the non-HEDU patient in 2018 and 2019, respectively (Table 2).

**Table 2:** Comparison of visit, patient and costs by HEDU type.

	All	Non-HEDU 1–3	HEDU > = 4			
				Repeat HEDU 4–7 visits	High HEDU 8–18 visits	Super HEDU > = 19 visits
2018–19 visits N (%)	121,403 (100.0%)	94,185 (77.5%)	27,218 (22.4%)	19,551 (16.1%)	5,878 (4.8%)	1,789 (1.5%)
2018 visits N (%)	60,329 (100.0%)	47,025 (77.8%)	13,304 (22.1%)	9,727 (16.1%)	2,724 (4.5%)	853 (1.4%)
2019 visits N (%)	61,074 (100.0%)	47,160 (77.2%)	13,914 (22.8%)	9,824 (16.1%)	3,154 (5.2%)	935 (1.5%)
2018–19 patients N (%)	69,984 (100.0%)	65,742 (93.9%)	4,242 (6.1%)	3.715 (5.3%)	485 (0.7%)	42 (0.1%)
2018 patients N (%)	40,559 (100.0%)	37,060 (91.4%)	3,499 (8.6%)	3,035 (7.5%)	422 (1.0%)	42 (0.1%)
2019 patients N (%)	29,425 (100.0%)	28,862 (97.5%)	743 (2.5%)	680 (2.3%)	63 (0.2%)	0 (0.0%)
2018–19 Visits/patient Median (IQR)	1 (1,2)	1 (1,2)	5 (4,7)	5 (4,6)	12 (10,16)	41 (33,54)
Provider Cost	335,441,813.00kr ($40,867,882.40)	260,257832.00kr ($31,707,992.45)	75,183,981.00kr ($9,159,889.96)	54,051,921.00kr ($6,585,307.69)	16,220,488.00kr ($1,976,190.71)	4,911,572.00 ($598.391.55)
Radiology Cost	64,859,650.86kr ($7,902,045.84)	52,028,268.30kr ($6,338,760.01)	12,831,382.56kr ($1,563,285.83)	9,587,622.06kr ($1,168,088.76)	2,717,681.80kr ($331,103.33)	526,078.70kr ($64,093.75)
Cost*	442,520,842.74kr ($53,913,641.83)	344,993,616.27kr ($42,031,607.25)	97,527,226.48kr ($11,882,034.58)	70,516,596.05kr ($8,591,248.45)	21,098,470.27kr ($2,570,489.93)	5,912,160.15kr ($720,296.21)
Provider Cost per Visit Mean (95% CI)	2,763.04 kr (2346.00, 2872.00) $336.63 (285,82, 349.80)	2763.26kr (2346.00, 2872.00) $336.66 (285,82, 349.80)	2,762.29kr (2346.00, 2872.00) $336.54 (285,82, 349.80)	2,764.66kr (2346.00, 2872.00) $336.83 (285,82, 349.80)	2,759.53 (2346.00, 2872.00) $336.20 (285,82, 349.80)	2,745.43 (2346.00, 2872.00) $334.48 (285,82, 349.80)
Radiology Cost per Visit Mean (95% CI)	534.25kr (0, 2837.80) $65.09 (0, 345.74)	552.41kr (0, 2837.80) $67.30 (0, 345.74)	471.43kr (0, 2837.80) $57.44 (0, 345.74)	490.39kr (0, 2837.80) $59.75 (0, 345.74)	462.35 (0, 2837.80) $56.33 (0, 345.74)	294.06kr (0, 2786.00) $35.83 (0.339.43)
Cost per Visit Mean [95%CI]	3,645.06kr [2,346.00, 6666.08] $444.09 (285.82, 812.15)	3,662.94kr [2,346.00, 6,271.08] $446.27 (285.82, 764.02)	3,583.19kr [2,346.00, 6,519.73] $436.55 (285.82, 794.32)	3,606.80kr [2,362.73, 6,594.26] $439.43 (287.86, 803.40)	3,589.04kr [2,346.00, 6,472.51] $437.26 (285.82, 788.57)	3,304.73kr [2,346.00, 5,941.31] $402.63 (285.82, 723.85)
Provider Cost per patient Mean (95% CI)	4,793.12kr (2726.00, 11,342.00) $583.96 (332.12, 1,381.83)	3,598,78kr (2346,00, 8470.00) $438.45 (285.82, 1,031,93	17,723.71kr (10,816.00, 34,040.00) $2159.33 (1,317.75, 3,659.86)	14,549.64kr (10,731.00, 22,304.00) $1,772.63 (1,307.39, 2,717.36)	33,444.31kr (21,924.00, 56,751.00) $4,074.62 (2,671.07, 6.914.14)	116,942.20kr (63,913.00, 168,331.00) $14,247.42 (7,786.71, 20,508.27)
Radiology Cost per patient Mean (95% CI)	926.78kr (0,4,550.00) $112.91 (0, 554.34)	791.40kr (0, 3,788.40) $96,42 (0, 461.55)	3,024.84kr (0, 11,172.00) $368.53 (0, $1,361.12)	2580.79kr (0, 9,478.00) $314.43 (0, 1,154.73)	5,603.47kr (0, 17,648.80) $682.69 (0, 2150.21)	12,525.68kr (0, 27,745.00) $1,526.04 (0, 3,380.26)
Cost per patient Mean (95% CI)	6,323.17kr (2,726.00, 16,706.00) $770.37 (332.12, 2,035.34)	5,247.69kr (2,726.00, 11,905.23) $639.34 (332.12, 1450.45)	22,990.86kr (11,322,27, 47,160.15) $2801.05 (1379.43, 5,745.66)	18,981.59kr (11,196.00, 32,517.47) $2312.58 (1364.04, 3961.70)	43,502.00kr (24,720.48, 76,750.98) $5,299.98 (3,011.77, 9,350.80)	140,765.70kr (66,046.55, 200,996.30) $17,149.91 (8,046.65, 24,487.98)

kr (Swedish krona).

$ (US Dollars, January 1, 2018, conversion rate).

*Excludes ambulance cost.

The Repeat, High and Super HEDU visits were more likely to be from males (OR 1.1, 1.1, 1.3, respectively), self-referred (OR 1.5, 2.7, 5.2), present with abdominal pain (OR 1.3, 1.3, 4.0), arrive by ambulance (OR 1.1, 1.2, 2.2), during the night (OR 1.1, 1.2, 1.4) and from the Örebro municipal region (OR 1.3, 1.5, 2.4). Compared to the non-HEDU, Repeat (OR 1.6) and High HEDU (OR 1.8) visits were more likely to be from the elderly. In contrast, the Super HEDU visits were more likely to be from adults (OR 4.3). Repeat (OR 1.2) and High HEDU (OR 1.4) visits were more likely to be assigned a high acuity triage score; however, the opposite was true for the Super HEDU visit (OR 1.0) (Table 3).

**Table 3:** Associations between demographic factors and HEDU visit type.

**Demographic Factors**	**Odds Ratios (CI)**		
	**Repeat HEDU** **4–7 visits vs.** **Non-HEDU**	**High HEDU** **8–18 visits vs.** **Non-HEDU**	**Super HEDU** **> = 19 visits vs.** **Non-HEDU**
Age Group: Children: 0–14 yo Adult: 15–64 yo Elderly: > = 65 yo	Ref 0.7 (0.7, 0.8)* 1.4 (1.4, 1.5)*	Ref 1.2 (1.1, 1.3)* 1.6 (1.5, 1.8)*	*No data 2.0 (1.7,2.3)* Ref
Sex (Male vs. Female)	1.1 (1.1, 1.1)*	1.1 (1.1, 1.2)*	1.4 (1.2, 1.5)*
Acuity (RETTS 1–2 vs. RETTS 3–5)	1.2 (1.2, 1.3)*	1.4 (1.3, 1.5)*	1.0 (0.9, 1.1)
Chief Complaint (Abdominal pain vs. No Abdominal Pain)	1.4 (1.3, 1.4)*	1.4 (1.3, 1.5)*	4.1 (3.7, 4.5)*
Municipal Regional Code (Örebro vs. Outside Örebro)	1.3 (1.3, 1.4)*	1.5 (1.4, 1.6)*	2.4 (2.1, 2.7)*
Referred From (Self vs. Outside Clinic)	1.4 (1.4, 1.5)*	2.6 (2.3, 3.1)*	5.2 (3.5, 7.7)*
Arrival Mode (Ambulance vs. Walk-In)	1.1 (1.0, 1.1)*	1.1 (1.0, 1.2)*	2.1 (1.9, 2.4)*
Triage Time Day (8:00–15:59) Evening (16:00–23:59) Night (0:00–7:59)	Ref 0.9 (0.9, 1.0)* 1.1 (1.0, 1.1)*	Ref 1.0 (0.9, 1.1) 1.2 (1.1, 1.3)*	Ref 1.4 (1.3, 1.6)* 1.4 (1.2, 1.6)*

RETTS (Rapid Emergency Triage and Treatment System).

P<0.05.

Repeat, High and Super HEDU visits were more likely to be assigned to the surgical zone (OR 1.1, 1.2, 3.2, respectively). The visit was less likely to receive a radiologic test (OR 0.7, 0.6, 0.4) or achieve the 4-hr time target (OR 0.8, 0.8, 0.6). Repeat and High HEDU visits were more likely to be admitted (OR 1.4, 1.4, respectively). Super HEDU visits were less likely to be admitted (OR 0.9) but more likely to leave without being seen (OR 3.3) (Table 4).

**Table 4:** Associations between HEDU Visit type and selected outcomes.

	Outcome Measures (Odds Ratio (CI))				
	Surgical vs. Non-Surgical Zone	Radiology vs. No Radiology	Admission vs. Discharge	LWBS vs. Not LWBS	EDLOS< = 4hrs
Non-HEDU Repeat HEDU High HEDU Super HEDU	Ref 1.1 (1.1, 1.1)* 1.2 (1.2, 1.3)* 3.2 (3.0, 3.6)*	Ref 0.7 (0.7, 0.8)* 0.6 (0.6, 0.6)* 0.4 (0.3, 0.4)*	Ref 1.4 (1.3, 1.4)* 1.4 (1.3, 1.4)* 0.9 (0.8, 1.0)*	Ref 1.1 (1.0, 1.2) 1.2 (1.0, 1.4)* 3.3 (2.8, 4.0)*	Ref 0.8 (0.8, 0.8)* 0.8 (0.8, 0.8)* 0.6 (0.5, 0.7)*

LWBS (Left Without Being Seen); EDLOS (Emergency Department Length of Stay).

## Discussion

There were HEDUs at ÖUH. A small number of ED patients accounted for a significant proportion of visits and costs. Provider costs were the main contributor of total costs. Although HEDUs accounted for a smaller proportion of total radiology costs and had a lower radiology cost per visit, the Super HEDU radiology cost per patient was 16 times higher than the non-HEDU patient. The reason why is that a small number of Super HEDU patients would receive a disproportionate number of radiographic investigations which would increase the mean radiology cost per patient. The Super HEDUs were different from the other HEDU types because they arrived by ambulance despite not being elderly, had high left-without-being-seen rates, were assigned lower acuity scores, received less radiology, were self-referred, arrived during evening-night hours and had longer ED length-of-stays. This suggests that the Super HEDU’s perceived urgency for seeking emergency care was greater than what which was determined in the ED. This suggests that the ED assessment did not address the Super HEDU’s needs which may have led to more visits.

According to a systematic review, HEDUs are about 5% of the ED population, but account for 21–28% of ED visits and associated costs. The review found that HEDU were more likely to be elderly, female and have a mental health diagnosis [5]. At ÖUH, we found similar HEDU proportions and higher prevalence in the elderly, but there were differences. ÖUH HEDU were more likely to be male and the Super HEDU were more likely to be of adult age. American, English and Canadian studies have also found higher odds of HEDU amongst elderly patients with mental or chronic illness [5,8,24,25]. We did not find the mental health characteristics for ÖUH HEDUs. An explanation is that ÖUH psychiatric patients attend a different ED. Consequently, HEDU findings from other studies may not be useful to ÖUH if it decides to design interventions tailored to its population [4].

In Ontario, Canada, 5% of high-cost users account for 61% of home care and hospital costs [26]. Because ÖUH HEDUs were more likely to be admitted, costs were likely underestimated. Furthermore, like other studies [24,25,27], we have found ÖUH HEDUs were more likely to arrive by ambulance. Since we did not collect ambulance costs, we underestimated the HEDU cost.

Our pilot study was based on a high-quality administrative database with minimal missing data and duplicates. Selection bias was unlikely since we did not extract a data sample but included all electronic registrations after exclusions were removed. The linking of Swedish administrative data is reproducible and efficient by using a unique personal identification number and ED encounter number. Since there is a national standard for costing with validation by the Örebro economic department, cost data are highly reliable. There were, however, limitations. We only studied a single hospital, limiting generalizability; however, this was a pilot feasibility study. On the other hand, our case costing methods can be used broadly. There was information bias. We found the electronic health record data could have been more comprehensive. Diagnostic labels were basic and did not provide enough detail for a fulsome analysis. Consequently, we only used the presenting complaint. There was no record of mental health or substance abuse in ÖUH administrative databases, although found in other Swedish studies [7]. The re-direction of psychiatric presentations to another ED was a contributing factor, but we suspect that ÖUH encountered these presentations without being recorded.

To further study HEDU in Sweden, administrative databases could be made more comprehensive. Not only could more diagnostic data be collected, but also socioeconomic [28], resource, community care, admission, costs and qualitative data [11] too. Although individual hospital HEDU populations are not necessarily similar, they could share data to create regional or national databases. Finding common HEDU characteristics could help develop prediction tools [29,30]. With readily available cost data, it is easier to perform economic analyses.

We suggest that HEDUs could be a health system performance indicator of coordinated care. The reason why is that interventions to decrease HEDU visits are coordinated care models, such as case management, individualized care plans and information sharing [31]. According to the OECD, Sweden’s healthcare system is highly integrated [32] but struggles to provide better care coordination for those with chronic diseases and access to primary care [33], so perhaps the HEDU population could be a marker of coordination.

The Institute of Healthcare Improvement’s “Triple Aim” for populations is: “1) Improving the patient experience of care 2) Improving the health of populations and 3) Reducing the per capita cost of health care. [34]” We suggest that HEDU costs be considered as a quality improvement measure for any healthcare system trying to achieve the Triple Aim [35]. In Ontario, Canada, Health Quality Ontario uses quality improvement with coordinated care plans, called *HealthLinks*, to improve the care for the HEDU and high-cost user population [36,37]. A 2015 systematic review found the impact of coordinated care plans, information sharing and case management to be modest but not ineffective [31].

Given the unique features of the ÖUH HEDU population, a local, tailored strategy is required. Consequently, a quality improvement plan would be a suitable approach [38]. Because of ÖUH’s large number of rotating physicians, it will be challenging to identify HEDUs through them. The electronic health record [39] and social workers [28] are potential identification strategies. For more effective community care, community health services, emergency department, paramedics, medical and surgical services could work together to develop coordinated care plans [40–43]. We recommend that coverage be extended into night hours and that coordinated care plans be tailored to the individual, requiring continual review [4].

### Conclusion

There is a HEDU population at ÖUH who account for a disproportionate amount of ED costs. From the electronic health record, there are factors and outcomes associated with being a HEDU that are unique to Örebro and not found within international literature. It is feasible to collect HEDU data from Swedish hospitals and municipal region databases.

## Supporting information

S1 Checklist (DOCX)
